# Long-Chain Acylcarnitines Decrease the Phosphorylation of the Insulin Receptor at Tyr1151 Through a PTP1B-Dependent Mechanism

**DOI:** 10.3390/ijms22126470

**Published:** 2021-06-16

**Authors:** Karlis Vilks, Melita Videja, Marina Makrecka-Kuka, Martins Katkevics, Eduards Sevostjanovs, Aiga Grandane, Maija Dambrova, Edgars Liepinsh

**Affiliations:** 1 Laboratory of Pharmaceutical Pharmacology, Latvian Institute of Organic Synthesis, Aizkraukles Str. 21, LV-1006 Riga, Latvia; melita.videja@farm.osi.lv (M.V.); makrecka@farm.osi.lv (M.M.-K.); maija.dambrova@farm.osi.lv (M.D.); ledgars@farm.osi.lv (E.L.); 2 Faculty of Biology, University of Latvia, Jelgavas Str. 1, LV-1004 Riga, Latvia; 3 Faculty of Pharmacy, Riga Stradins University, Dzirciema Str. 16, LV-1007 Riga, Latvia; 4 Biologically Active Compound Synthesis Laboratory, Latvian Institute of Organic Synthesis, LV-1006 Riga, Latvia; martins@osi.lv; 5 Laboratory of Physical Organic Chemistry, Latvian Institute of Organic Synthesis, LV-1006 Riga, Latvia; eduards@osi.lv; 6 Organic Synthesis Group, Latvian Institute of Organic Synthesis, LV-1006 Riga, Latvia; aiga@osi.lv

**Keywords:** palmitoylcarnitine, protein tyrosine phosphatase 1B, long-chain acylcarnitines, Akt, insulin receptor, insulin resistance

## Abstract

The accumulation of lipid intermediates may interfere with energy metabolism pathways and regulate cellular energy supplies. As increased levels of long-chain acylcarnitines have been linked to insulin resistance, we investigated the effects of long-chain acylcarnitines on key components of the insulin signalling pathway. We discovered that palmitoylcarnitine induces dephosphorylation of the insulin receptor (InsR) through increased activity of protein tyrosine phosphatase 1B (PTP1B). Palmitoylcarnitine suppresses protein kinase B (Akt) phosphorylation at Ser473, and this effect is not alleviated by the inhibition of PTP1B by the insulin sensitizer bis-(maltolato)-oxovanadium (IV). This result indicates that palmitoylcarnitine affects Akt activity independently of the InsR phosphorylation level. Inhibition of protein kinase C and protein phosphatase 2A does not affect the palmitoylcarnitine-mediated inhibition of Akt Ser473 phosphorylation. Additionally, palmitoylcarnitine markedly stimulates insulin release by suppressing Akt Ser473 phosphorylation in insulin-secreting RIN5F cells. In conclusion, long-chain acylcarnitines activate PTP1B and decrease InsR Tyr1151 phosphorylation and Akt Ser473 phosphorylation, thus limiting the cellular response to insulin stimulation.

## 1. Introduction

Insulin signalling plays a central role in the regulation of glucose and lipid metabolism. The activation of insulin signalling begins when insulin binds to the extracellular subunits of the insulin receptor (InsR), causing conformational changes that promote the autophosphorylation of intracellular subunits, including the Tyr1151 phosphorylation site [1]. This step of insulin signalling is regulated by tyrosine phosphatases; among these tyrosine phosphatases, the most noteworthy is protein-tyrosine phosphatase 1B (PTP1B, also referred to as PTPN1), which is considered the main regulator of InsR phosphorylation [2]. Activated PTP1B catalyses the dephosphorylation of InsR, thus downregulating insulin signal transduction [3]. After the sequential phosphorylation events mediated by downstream kinases, insulin signalling reaches protein kinase B (Akt), a central member of the insulin signalling pathway. Akt is activated when it binds to the cellular membrane [4,5], exposing two main phosphorylation sites, namely, Tyr308 and Ser473. Akt phosphorylation is regulated by ceramide-activated protein phosphatase 2A (PP2A) [6]. In addition, multiple diacylglycerol (DAG)-dependent isoforms of protein kinase C (PKC) are able to regulate Akt phosphorylation [7]. Insulin-stimulated Akt phosphorylation at Ser473 initiates physiological processes associated with facilitating glucose uptake into the cell by glucose transporter type 4 [8] and limiting fatty acid metabolism by inhibiting carnitine palmitoyltransferase 1 (CPT1)-mediated long-chain acylcarnitine (LCAC) synthesis [9,10].

Insulin resistance is associated with increased levels of lipid metabolism intermediates, such as DAG, ceramides, and long-chain fatty acids and their acylcarnitines [11]. The role of LCAC in the regulation of intracellular signalling pathways and the contribution of LCAC to insulin resistance have received relatively little attention. To date, researchers have determined that LCAC dictates energy metabolism patterns in mitochondria by inhibiting pyruvate-lactate oxidation [12,13]. Increased levels of LCAC in mice induce marked hyperinsulinaemia, insulin resistance, and glucose intolerance [14]. The role of LCAC in the regulation of insulin signalling has been highlighted in studies showing that LCAC induces a decrease in Akt phosphorylation in ex vivo [15,16] and in vivo [14] models, although the exact molecular mechanism has not yet been defined.

In this study, we selected the CHO INSR 1284 cell line (CHO), which continuously overexpresses human InsR [17]. Because the expression of InsR in CHO cells is higher than that in most cell cultures, this cell line is an appropriate system for studies of the regulation of metabolism [17]. As shown in our previous study, a highly abundant LCAC, palmitoylcarnitine, increases the concentration of circulating insulin in vivo [14]. We used the insulin-secreting cell line RIN5F to further investigate the effects of LCAC on insulin release [18]. Insulin-sensitizing bis-(maltolato)-oxovanadium (IV) (BMOV) and the non-competitive PTP1B inhibitor 4-hydroxy-3,3-dimethyl-2*H*-benzo[*g*]indole-2,5(3*H*)-dione (BVT948) were used as inhibitors of PTP1B [19,20]. In addition, a zinc-containing compound (zinc sulfate) was used as an inhibitor of PP2A [21], and sotrastaurin was used as an inhibitor of both conventional and novel DAG-dependent PKC isoforms [22].

## 2. Results

### 2.1. Palmitoylcarnitine Diminishes Insulin-Stimulated Phosphorylation of InsR and Akt

**Figure 1:**
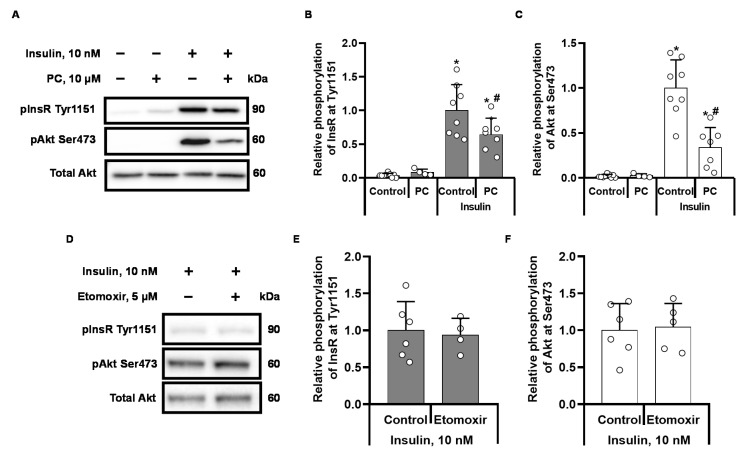
Palmitoylcarnitine (PC) and etomoxir altered the phosphorylation of InsR and Akt in insulin-stimulated CHO InsR 1284 cells. CHO cells were incubated in DMEM low glucose media with and without 10 nM insulin and 10 µM palmitoylcarnitine for 10 min. Representative bands from insulin-stimulated cells (**A**,**D**). Semiquantitative data showing the relative phosphorylation of InsR at Tyr1511 (**B**,**E**) and Akt at Ser473 (**C**,**F**). The band intensities were normalized by dividing each value by the intensity of the total Akt or InsR band. The band intensities of the insulin control were set to 1. * *p* < 0.05 compared to the unstimulated control group and # *p* < 0.05 compared to the insulin-stimulated group (one-way ANOVA, Tukey’s multiple comparison test). The results are presented as the means ± standard deviation. Sample size of the groups in sections B and C: control *n* = 9, PC *n* = 4, insulin control *n* = 8, and insulin + PC *n* = 7. Sample size of groups in section E: insulin *n* = 6, insulin + etomoxir *n* = 4. Sample size of groups in section F: insulin *n* = 6, insulin + etomoxir *n* = 5.

Stimulation of the CHO INSR 1284 cell line (ATCC^®^ CRL-3307™) with 10 nM insulin significantly increased the phosphorylation of InsR at Tyr1151 by 25-fold (Figure 1A,B) and the phosphorylation of Akt at Ser473 by approximately 50-fold (Figure 1A,C), indicating subsequent amplification of the insulin signal. When cells were stimulated with 10 nM insulin in the presence of 10 µM palmitoylcarnitine, the insulin-induced phosphorylation of InsR at Tyr1151 was decreased by 35 ± 24% (*p* < 0.05) compared to cells stimulated with insulin alone (Figure 1B). Compared to insulin alone, the addition of palmitoylcarnitine to the cell culture media also resulted in a subsequent reduction in Akt Ser473 phosphorylation by 66 ± 22% (*p* < 0.005) (Figure 1C). These findings suggest that palmitoylcarnitine affects both InsR phosphorylation and Akt Ser473 phosphorylation. The baseline levels of InsR Tyr1151 and Akt Ser473 phosphorylation in unstimulated CHO cells were very low, and the addition of 10 µM palmitoylcarnitine to the CHO cell media did not cause measurable changes in Akt phosphorylation (Figure 1A,C). Treatment with the CPT1 inhibitor etomoxir did not affect insulin-stimulated InsR and Akt phosphorylation (Figure 1D–F), indicating that endogenous acylcarnitine synthesis in CHO cells does not produce a sufficient amount of LCACs to affect insulin signalling.

### 2.2. Inhibition of PTP1B Alleviates the Palmitoylcarnitine-Induced Dephosphorylation of InsR Tyr1151

**Figure 2:**
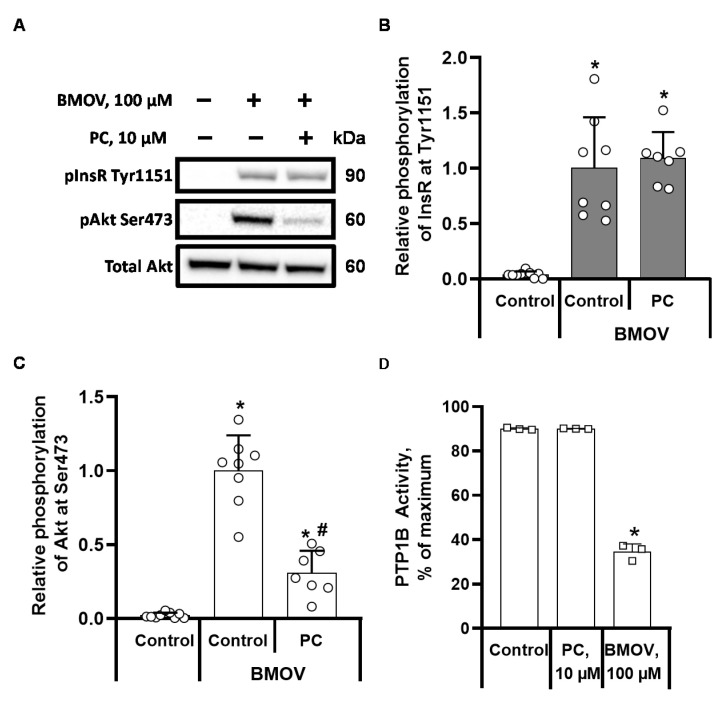
Effects of bis-(maltolato)-oxovanadium (IV) (BMOV) on the phosphorylation of InsR and Akt in CHO InsR 1284 cells and on the activity of purified protein tyrosine phosphatase 1B (PTP1B). CHO cells were preincubated with 100 µM BMOV for 1 h, and 10 µM palmitoylcarnitine (PC) was added 10 min before the cells were harvested for WB. Representative bands from BMOV-stimulated cells are shown (**A**). Semiquantitative data showing the relative phosphorylation of InsR at Tyr1511 (**B**) and Akt at Ser473 (**C**). Activity of purified PTP1B in the presence of PC and BMOV (**D**). The band intensities were normalized by dividing each value by the intensity of the total Akt or InsR band. The band intensities of the BMOV control were set to 1. * *p* < 0.05 compared to the unstimulated control group and # *p* < 0.05 compared to the BMOV-stimulated group (one-way ANOVA, Tukey’s multiple comparison test). The results are presented as the means ± standard deviation. Sample size of groups in sections B and C: control *n* = 9, BMOV control *n* = 8, and BMOV + PC *n* = 7. Sample size of groups in section D: control *n* = 3, PC *n* = 3, and BMOV *n* = 3.

**Figure 3:**
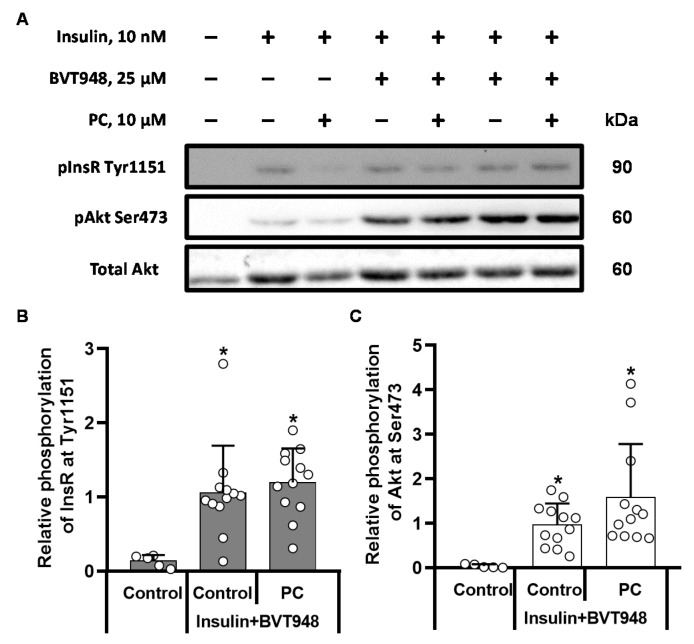
Effects of BVT948 on the palmitoylcarnitine (PC)-induced changes in InsR and Akt phosphorylation in insulin-stimulated CHO InsR 1284 cells. CHO cells were preincubated with 25 µM BVT948 for 1 h, and 10 µM PC and 10 nM insulin were added 10 min before the cells were harvested for WB. Representative bands show InsR and Akt phosphorylation (**A**). Semiquantitative data showing the relative phosphorylation of InsR at Tyr1511 (**B**) and Akt at Ser473 (**C**). The band intensities were normalized by dividing each value by the intensity of the total Akt or InsR band. The band intensities of the insulin-BVT948-stimulated control were set to 1. * *p* < 0.05 compared to the unstimulated control group (Kruskal-Wallis test, Dunn’s multiple comparison test). The results are presented as the means ± standard deviation. Sample size of groups: control *n* = 5, insulin + BVT948 *n* = 12, and BVT948 + PC *n* = 12.

We used the insulin sensitizer vanadium-containing compound BMOV, which is known to increase InsR phosphorylation through PTP1B inhibition, to determine whether the effect of palmitoylcarnitine on the dephosphorylation of InsR was related to PTP1B. In CHO cells stimulated with 100 µM BMOV, the levels of InsR Tyr1151 and Akt Ser473 phosphorylation (Figure 2A) were similar to those observed in insulin-stimulated cells (Figure 1A). We observed 25-fold and 50-fold BMOV-stimulated increases in InsR Tyr1151 (Figure 2B) and Akt Ser473 (Figure 2C) phosphorylation, respectively. The addition of 10 µM palmitoylcarnitine to BMOV-stimulated cells did not induce any decrease in the phosphorylation of InsR at Tyr1151 (Figure 2B). However, despite the preserved phosphorylation of InsR, the level of Akt Ser473 phosphorylation in palmitoylcarnitine-treated cells was significantly decreased by 69 ± 15% (*p* < 0.005) (Figure 2C) compared to that in BMOV-stimulated cells. Based on these results, palmitoylcarnitine influences InsR through the activation of PTP1B, and palmitoylcarnitine reduces the level of Akt phosphorylation independent of its effect on InsR. Since the palmitoylcarnitine-induced dephosphorylation of InsR at Tyr1151 was abolished after the inhibition of PTP1B by BMOV, we determined whether palmitoylcarnitine induced the dephosphorylation of InsR through the direct activation of the tyrosine phosphatase PTP1B. We tested the effect of palmitoylcarnitine in a purified tyrosine phosphatase PTP1B enzyme activity assay. At a concentration of 100 µM, BMOV decreased PTP1B activity by 62 ± 4% (Figure 2D), but 10 µM palmitoylcarnitine did not cause any changes in PTP1B activity. Therefore, palmitoylcarnitine indirectly activates PTP1B and stimulates the dephosphorylation of InsR at Tyr1151. We used the non-competitive PTP1B inhibitor BVT948 together with insulin to confirm these results. BVT948 completely blocked palmitoylcarnitine-induced dephosphorylation of InsR and Akt (Figure 3A–C), supporting the hypothesis that PTP1B is a target of LCAC action.

**Figure 4:**
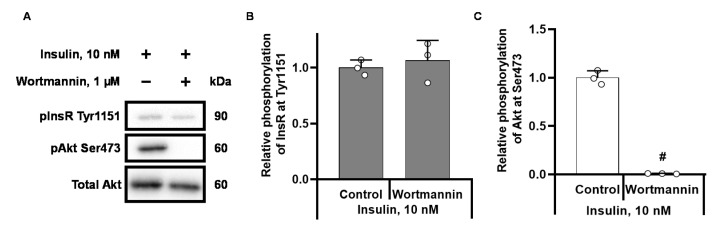
Effects of wortmannin on the phosphorylation of InsR (**A**,**B**) and Akt (**A**,**C**) in insulin-stimulated CHO InsR 1284 cells. The band intensities were normalized by dividing each value by the intensity of the total Akt or InsR band. The band intensities of the insulin-stimulated control were set to 1. # *p* <0.05 compared to the insulin control group (one-way ANOVA, Tukey’s multiple comparison test). The results are presented as the means ± standard deviation. Sample size of groups: insulin *n* = 3, insulin + wortmannin *n* = 3.

Wortmannin did not affect insulin-stimulated phosphorylation of InsR at Tyr1151 (Figure 4A,B) but completely prevented Akt Ser473 phosphorylation (Figure 4A,C).

### 2.3. Palmitoylcarnitine Decreases Akt Phosphorylation Independently of PP2A and PKC Activity

**Figure 5:**
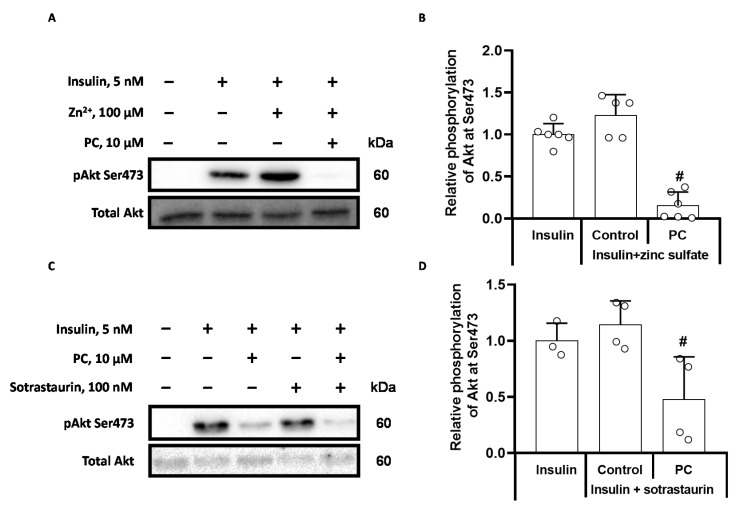
Effects of the PP2A inhibitor Zn^2+^ and the PKC inhibitor sotrastaurin on palmitoylcarnitine-induced Akt phosphorylation in insulin-stimulated CHO InsR 1284 cells. CHO cells were preincubated in DMEM low glucose media containing 100 µM zinc sulfate (**A**) or 100 nM sotrastaurin (**C**) for 50 min and then stimulated with 5 nM insulin for 10 min in the presence or absence of 10 µM palmitoylcarnitine (PC). Representative bands from cells treated with zinc sulfate (**A**) or 100 nM sotrastaurin (**C**). Semiquantitative data showing the relative phosphorylation of Akt at Ser473 (B and D). The band intensities were normalized by dividing each value by the intensity of the total Akt band. The band intensities of the insulin control were set to 1. Quantification of relative Akt phosphorylation and PC-induced effects in the presence of zinc sulfate (**B**) and sotrastaurin (**D**) is shown. * *p* < 0.05 compared to the unstimulated control group and # *p* < 0.05 compared to the insulin/Zn^2+^-stimulated group (one-way ANOVA, Tukey’s multiple comparison test). The results are presented as the means ± standard deviation. Sample size of the groups in section B: insulin *n* = 6, Zn^2+^ + insulin *n* = 5, and PC + Zn^2+^ + insulin *n* = 6; section D: insulin *n* = 3, sotrastaurin + insulin *n* = 4 and PC + sotrastaurin + insulin *n* = 4.

We used sotrastaurin and zinc sulfate, which are inhibitors of PKCs and PP2A, to determine whether the effects of palmitoylcarnitine on Akt phosphorylation were mediated by these enzymes. Stimulation of the cells with insulin in the presence of 100 µM zinc sulfate (Figure 5A,B) and 100 nM sotrastaurin (Figure 5C,D) did not exert a significant effect on Akt Ser473 phosphorylation compared to stimulation with insulin alone. When palmitoylcarnitine was added to the insulin- and Zn^2+^-stimulated cells, the level of Akt Ser473 phosphorylation was completely diminished (Figure 5A,B). This finding indicates that palmitoylcarnitine does not induce the dephosphorylation of Akt Ser473 through PP2A (Figure 5B). A similar effect was observed when sotrastaurin was used. Since 100 nM sotrastaurin did not alleviate the effects of palmitoylcarnitine on insulin stimulation (Figure 5D), DAG-dependent PKC isoforms are likely not involved in the palmitoylcarnitine-induced dephosphorylation of Akt. Taken together, palmitoylcarnitine induces Akt Ser473 dephosphorylation independently of PP2A and PKC activity.

### 2.4. Palmitoylcarnitine Increases Insulin Release by Limiting Akt Ser473 Phosphorylation in Insulin-Producing RIN5F Cells

**Figure 6:**
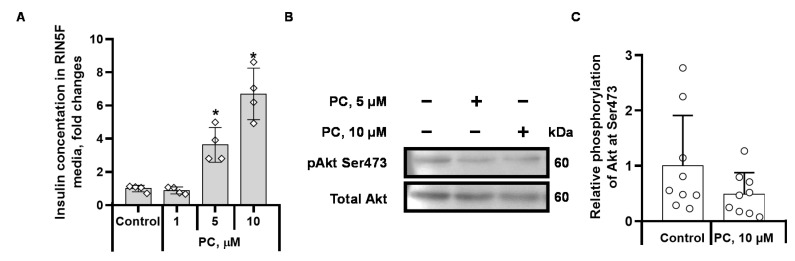
Effects of palmitoylcarnitine (PC) on insulin-producing RIN5F cells. Insulin release in the presence of PC (**A**). The insulin secretion in the media was compared by normalizing the insulin concentration to that of the control group incubated without PC. The graph shows the relative insulin concentration compared to the control group treated without PC. Insulin release was measured 1 h after the cell growth media was changed to DMEM high glucose media (11 mM glucose) containing various concentrations of PC. Representative bands from RIN5F cells (**B**). Semiquantitative data showing relative Akt Ser473 phosphorylation (**C**). The phosphorylation of Akt at Ser473 in RIN5F cells was determined using WB after a 1 h incubation with various concentrations of PC. The band intensities were normalized by dividing each value by the intensity of the total Akt band. The band intensities of the control were set to 1. The results are presented as the means ± standard deviation. * *p* < 0.05 compared to the control group (one-way ANOVA, Tukey’s multiple comparisons test). Sample size of the groups in section A: control *n* = 4, PC 1 µM *n* = 4, PC 5 µM *n* = 4, and PC 10 µM *n* = 4. Sample size of the groups in section C: control *n* = 9, PC 10 µM *n* = 9.

**Figure 7:**
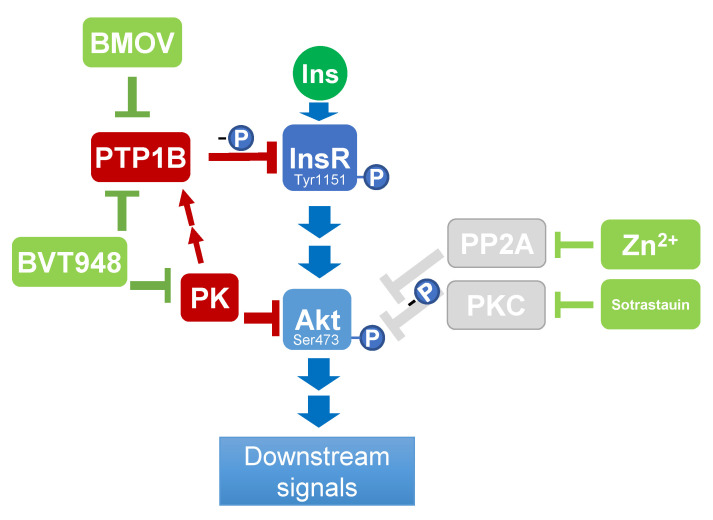
Summary of the effects of palmitoylcarnitine (PC) on cells. PC inhibits insulin signalling by decreasing the phosphorylation of InsR and Akt. PC does not decrease the phosphorylation of InsR if it is stimulated by the PTB1B inhibitor bis-(maltolato)-oxovanadium (IV) (BMOV). However, PC still markedly reduces the phosphorylation of the downstream target Akt in the presence of BMOV. Abbreviations and symbols: blue—normal physiological response, red—PC-induced changes. InsR—insulin receptor β, PTP1B—protein-tyrosine phosphatase 1B, Akt—protein kinase B, Ins—insulin, “-P”—dephosphorylation, and “+P”—phosphorylation.

We used the insulin-producing RIN5F cell line and measured the levels of Akt Ser473 phosphorylation and insulin release in the presence of palmitoylcarnitine to elucidate the possible mechanisms underlying LCAC-induced hyperinsulinaemia. When RIN5F cells were incubated in cell media containing palmitoylcarnitine for 1 h, a dose-dependent increase in insulin release was observed (Figure 6A). At a 1 µM concentration, palmitoylcarnitine did not affect insulin release, while palmitoylcarnitine applied at concentrations of 5 µM and 10 µM stimulated an increase in the insulin concentration by 3.6- and 6.7-fold, respectively, compared to the control (Figure 6A). Despite the approximately 7-fold increase in the insulin concentration, we did not observe a statistically significant increase in Akt Ser473 phosphorylation (Figure 6B,C), indicating that despite the high insulin concentration, palmitoylcarnitine hindered the ability of the cells to detect insulin through the inhibition of Akt Ser473 phosphorylation (Figure 6B,C and Figure 7), and a higher concentration of insulin is required to prevent insulin release.

## 3. Discussion

**Figure 8:**
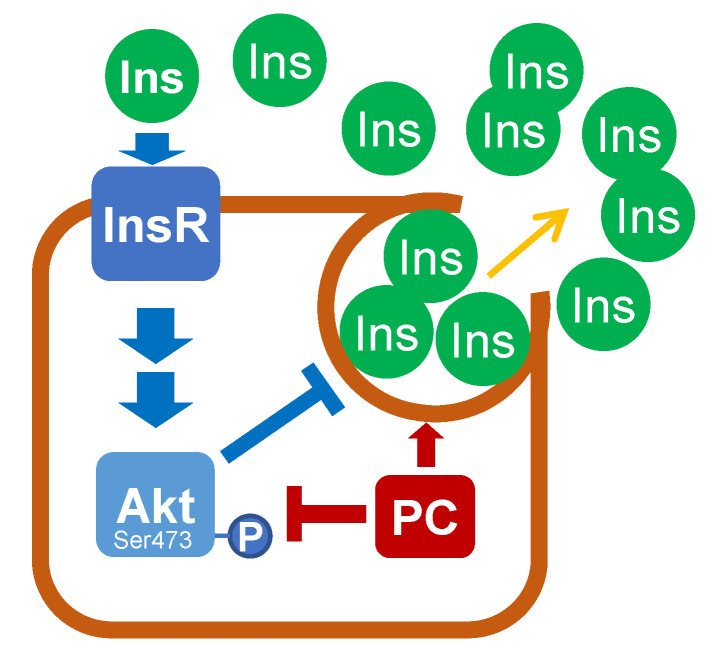
Effects of palmitoylcarnitine (PC) on insulin release. By decreasing Akt phosphorylation in the RIN5F cell line, PC interferes with the insulin-sensing feedback mechanism, thus promoting excessive insulin release. Abbreviations and symbols: blue—normal physiological response, red—PC-induced changes. InsR—insulin receptor β, Akt—protein kinase B, Ins—insulin, “-P”—dephosphorylation, and “+P”—phosphorylation.

Here, we showed for the first time that palmitoylcarnitine affects the insulin signalling pathway by inducing the dephosphorylation of InsR at Tyr1151 (Figure 7). The effects of palmitoylcarnitine on the dephosphorylation of InsR at Tyr1151 were related to the indirect activation of PTP1B. Additionally, palmitoylcarnitine significantly decreased the insulin- and BMOV-stimulated phosphorylation of Akt at Ser473. Thus, the inhibitory effect of palmitoylcarnitine on Akt activity is independent of the upstream effects on InsR phosphorylation. The inhibition of PP2A and PKCs did not diminish the palmitoylcarnitine-induced decrease in Akt Ser473 phosphorylation, indicating that PP2A and PKC are not involved in the palmitoylcarnitine-induced dephosphorylation of Akt. Based on these results, palmitoylcarnitine affects insulin signalling pathways through different mechanisms from ceramide and DAG. Finally, our results show that the molecular origins of insulin resistance and hyperinsulinaemia, which are observed in insulin-resistant individuals, are potentially explained by the effects of LCAC on the insulin signalling pathway in insulin-secreting cells (Figure 8).

The physiological importance of PTP1B in the insulin signalling pathway was previously established by the findings that the deletion of PTP1B results in insulin hypersensitivity [23] and that overexpression of PTP1B causes insulin resistance, similar to that observed in patients with type 2 diabetes [24]. A novel finding in this study is that palmitoylcarnitine decreases the phosphorylation of InsR at Tyr1151 through a PTP1B-dependent mechanism. Moreover, palmitoylcarnitine does not decrease the phosphorylation of InsR in cells that are treated with the PTB1B inhibitors BMOV and BVT948, suggesting that indirect PTB1B activation is the main mechanism by which palmitoylcarnitine acts on InsR. Because PTP1B is a crucial component of the insulin signalling pathway, the regulation of PTP1B activity is a complex process that involves numerous stages and feedback loops, and this process might be affected by palmitoylcarnitine. Akt plays a central role in the insulin signalling pathway, and its activity is a measure of insulin sensitivity [25]. Akt Ser473 phosphorylation induces numerous insulin-dependent effects both in vitro and in vivo. In this study, we observed a pronounced decrease in Akt Ser473 phosphorylation after stimulation with insulin or BMOV. Since the inhibition of PTB1B fully restores the phosphorylation of InsR, we conclude that palmitoylcarnitine decreases Akt phosphorylation independently of InsR. Importantly, the effects of LCAC on insulin signalling induce changes in insulin-dependent glucose metabolism [14,15]. In addition to mediating the insulin response in a cell, Akt serves as a positive regulator of InsR by phosphorylating PTP1B at the Ser50 position, thus hindering its phosphatase activity [26] and indirectly increasing InsR phosphorylation. By decreasing Akt activity, palmitoylcarnitine compromises the Akt-dependent regulation of PTP1B activity (Figure 7), resulting in a higher InsR dephosphorylation rate. Overall, the palmitoylcarnitine-induced decrease in InsR phosphorylation might at least partially depend on the palmitoylcarnitine-induced decrease in Akt Ser473 phosphorylation. However, wortmannin treatment did not cause significant changes in InsR Tyr1151 phosphorylation (Figure 4B), suggesting that the Akt-PTP1B regulatory mechanism does not explain palmitoylcarnitine-induced effects on the CHO cell line.

We used BVT948, a non-competitive, irreversible inhibitor of the protein tyrosine phosphatases PTP1B (IC50 0.9 µM), T-cell protein tyrosine phosphatase (TCPTP) (IC50 1.7 µM), tyrosine-protein phosphatase nonreceptor type 11 (SHP-2) (IC50 0.09 µM), and leukocyte common antigen-related protein tyrosine phosphatase (LAR) (IC50 1.5 µM) to further clarify the effects of palmitoylcarnitine [20]. BVT948 preserves InsR and Akt Ser473 phosphorylation in the presence of palmitoylcarnitine; therefore, all kinases in the insulin signalling pathway are not inhibited by palmitoylcarnitine. Thus, the palmitoylcarnitine mechanism of action is related to the activation of BVT948-targeted phosphatases. Furthermore, the effect of palmitoylcarnitine on Akt phosphorylation is not diminished by BMOV, suggesting that palmitoylcarnitine-induced effects on Akt may be mediated by phosphatases that are unaffected by BMOV but inhibited by BVT948 [20,27]. Overall, two phosphatases, LAR [28] and TC-PTP [29], are most likely affected by palmitoylcarnitine, as both are described as negative regulators of the insulin signalling pathway.

The physiological significance of PP2A and its involvement in insulin signalling have been revealed by the findings that PP2A deletion leads to insulin hypersensitivity [30] but PP2A overexpression induces insulin resistance [24]. PP2A has been described as a negative regulator of Akt Tyr308 phosphorylation, but recent studies have also indicated its involvement in the regulation of Akt Ser473 phosphorylation [31]. In this study, PP2A inhibition by zinc did not alleviate the effects of palmitoylcarnitine on CHO cells, indicating that palmitoylcarnitine does not decrease Akt phosphorylation through the same mechanism as ceramides. The insulin-sensitizing and mimetic effects of Zn^2+^ are attributed not only to the inhibition of PP2A [32] but also to the inhibition of multiple other phosphotyrosine phosphatases [33], including phosphatase and tensin homologue (PTEN) [34], which are negative regulators of the Akt signalling pathway. Thus, since the Zn^2+^ treatment did not alter the effects of palmitoylcarnitine on Akt Ser473 phosphorylation, we conclude that PP2A and PTEN are not involved in the effects of palmitoylcarnitine on insulin signalling (Figure 7).

The PKC family participates in the regulation of the insulin signalling pathway by influencing Akt phosphorylation. Increased expression and activity of PKC isoforms are associated with impaired insulin signalling in subjects with insulin resistance [22,35]. Due to the high similarity between the different PKC isoforms, we used the nonspecific inhibitor sotrastaurin, which is a compound that inhibits most of the DAG-dependent PKC isoforms, including α, βI, δ, ε, η, and θ [36]. Incubation of the cells with sotrastaurin did not result in any changes in the insulin-stimulated phosphorylation of Akt at Ser473 and did not alleviate the effects of palmitoylcarnitine. These results indicate that palmitoylcarnitine affects the insulin signalling pathway through a mechanism that differs from PKC activation by DAGs.

The mechanism underlying the autocrine control of insulin release from β-cells has been extensively studied for many years. First, Leibiger et al. noted that phosphoinositide 3-kinase is required for the autocrine control of insulin synthesis in islet β-cells [37]. Subsequent studies elucidated that Akt controls the rate of insulin release, and alterations in Akt signalling result in dysfunctional insulin release from β-cells [38]. The accumulation of LCAC increases insulin secretion from β-cells in vitro [39] and the overall concentration of circulating insulin in vivo [14]. In the present study, we observed lower Akt Ser473 phosphorylation in RIN5F cells treated with palmitoylcarnitine, despite the increased insulin release and the corresponding increased concentration of insulin in the cell media. Thus, palmitoylcarnitine-stimulated insulin release is most likely caused by palmitoylcarnitine-induced suppression of Akt Ser473 phosphorylation (Figure 8).

Our results further confirm recent findings showing that LCAC interacts with insulin signalling [14,15,16,40,41]. In this study, we documented that the lower level of Akt Ser473 phosphorylation is at least partially caused by LCAC-stimulated PTP1B-dependent dephosphorylation of InsR. In a study by Pereyra et al. using a mouse model with muscle-specific CPT2 knockout (Sk^−/−^), an increased content of LCAC in muscle did not result in an impairment in insulin and glucose tolerance in vivo [42]. Notably, neither glucose uptake nor the oxidation rate was assessed in the muscles of Sk^−/−^ mice, which would be important for in vivo studies where multiple tissues are involved in insulin-dependent glucose uptake. Nevertheless, increased levels of LCAC significantly reduced Akt Ser473 phosphorylation in liver and adipose tissue, as well as muscle from HFD-treated mice [42]. These inhibitory effects of LCAC accumulation on Akt Ser473 phosphorylation are consistent with our present study.

Since Randle et al. proposed the competition of lipid and glucose oxidative flux [43], extensive research has been conducted to fully comprehend all aspects of lipid and glucose metabolite crosstalk and their regulatory functions [44]. The role of these interactions has become particularly relevant for understanding the mechanisms underlying insulin resistance, which precedes the onset of type 2 diabetes. An elevated LCAC content is associated with impaired insulin sensitivity in patients [15], and this association has been further characterized in animal models of insulin resistance [45,46]. Our present data highlight a role for LCAC in the regulation of the molecular mechanisms of insulin signalling and explain the aspects of insulin resistance and hyperinsulinaemia, which are associated with type 2 diabetes. Therefore, the results of this study suggest that reducing LCAC levels might represent a novel treatment strategy for improving insulin sensitivity.

## 4. Materials and Methods

### 4.1. CHO INSR 1284 Cell Culture

CHO INSR 1284 cells (ATCC^®^ CRL-3307™) were obtained from ATCC^®^ (Lielpin, Lomianki, Poland). The cells were grown in Ham’s F-12 medium with Glutamax (cat. 31765-035, Thermo Fisher Scientific, Waltham, MA, USA) supplemented with 10% *v*/*v* heat-inactivated foetal bovine serum (FBS) (cat. F7524, Merck KGaA, Darmstadt, Germany) and 0.34 mg/mL hygromycin B (cat sc-29067, Santa Cruz Biotechnology, Heidelberg, Germany) at 37 °C in a HERAcell VIOS 160i CO_2_ incubator (Thermo Fisher Scientific) with 5% CO_2_. The cell cultures were maintained by subculturing the cells every 2–3 days. Before the experiments, 1 mL of a cell suspension was plated in TC 24-well Cell+, F plates (cat, 83.3922.300, Starstedt, Nümbrecht, Germany) at a density of 10^5^ cells/mL and incubated overnight. Before the experiment, the cell media were changed to DMEM low glucose (cat. D5523, Merck KGaA) without FBS. The compounds or the respective vehicle controls were added and incubated with the cells. After incubation, the cell media were removed completely, and lysis buffer was added. All cell experiments were performed in serum- and BSA-free media; however, our data indicate that the addition of BSA to cell media does not change the palmitoylcarnitine-induced effect on insulin signalling (data not shown).

### 4.2. RIN5F Insulin Release Assay

RIN-5F cells (ATCC^®^ CRL-2058™) were obtained from ATCC^®^ and grown in 100 μL of RPMI 1640 medium (cat. 61870010, Thermo Fisher Scientific) supplemented with 10% *v*/*v* heat-inactivated FBS at a density of 2 × 10^5^ cells/mL. Twenty-four hours before the assay, the media were changed to DMEM high glucose (cat. D1152, Merck KGaA) without FBS. Immediately before the assay, the media were changed again to 100 μL of DMEM high glucose containing various concentrations of palmitoylcarnitine (0–10 μM). After 1 h, the cell media were collected and centrifuged at 380 g to safely remove any floating cells. The supernatant was frozen at −80 °C until further analysis. Insulin concentrations were measured using an insulin detection kit (Cat. No. EZRMI-13K, Merck KGaA). All cell experiments were performed in serum- and BSA-free media.

### 4.3. Tests for Cell Culture Contamination

All the cell lines used in this study were routinely tested for mycoplasma contamination using the MycoProbe Mycoplasma Detection Kit (CUL001B, R&D Systems, Inc., Minneapolis, MN, USA) according to the manufacturer’s protocol. Bacterial and fungal contaminations were assessed by visually inspecting the cell media in each well using a light microscope before performing the assay procedures.

### 4.4. The Protein Concentrations

Total protein concentration in the cell lysate was measured using the Lowry method with bovine serum albumin (BSA) (cat. EQBAH66, Europa Bioproducts Ltd., Chelsworth, UK) as the standard.

### 4.5. Preparation and Analysis of Protein Extracts Using Western Blotting

Protein extracts from CHO INSR 1284 cell cultures were prepared using a previously described method [14] with some modifications. Briefly, the cells were homogenized in urea buffer (4 mM urea (cat, U5378, Merck KGaA), 140 mM Tris base (cat, 648310, Merck KGaA), 1% SDS (cat. L3771, Merck KGaA), 1 mM glycerol 2-phosphate (cat. G9891, Merck KGaA), 1 mM NaF (cat. 60-013-87, Fisher Scientific Company, Pittsburgh, PA, USA), 10 μM leupeptin (cat 1167, Tocris Bioscience, Abingdon, UK), 1 μM pepstatin (cat 1190, Tocris Bioscience), 1 μM aprotinin (cat. A1153 Merck KGaA), and 100 μM AEBSF (cat. A8456, Merck KGaA)) for 20 s with an ultrasonic processor (Cole-Parmer, Vernon Hills, IL, USA) at a 20% amplitude. The samples were frozen at −80 °C or used immediately. The cell lysates were mixed with 6× Laemli buffer (12% *w*/*v* SDS, 60 mM Tris pH 6.8, 47% glycerol (cat. G7886, Merck KGaA), 0.93% *w*/*v* DTT (cat. 3154 Tocris Bioscience), and 0.06% *w*/*v* bromophenol blue (cat. 15613840, Fisher Scientific Company)), and 20 µg of total protein were loaded into each well of the gel. SDS-polyacrylamide gel electrophoresis (SDS-PAGE) gels consisting of a 2% stacking gel and an 8% resolving gel were cast in Invitrogen™ Bolt™ Empty Mini Gel Cassettes (cat. NW2010 Thermo Fisher Scientific). Electrophoresis was performed using a Thermo Scientific electrophoresis system (Thermo Fisher Scientific). Then, the proteins were transferred to Invitrogen™ iBlot™ 2 Transfer Stack PVDF membranes (cat. IB24001, Thermo Fisher Scientific) using the iBlot 2 Dry Blotting System (Thermo Fisher Scientific). After transfer, the polyvinylidene fluoride (PVDF) membranes were blocked with 5% BSA dissolved in TBS buffer (150 mM NaCl (S7653, Merck KGaA) and 50 mM Tris, pH 7.6) for 1 h at room temperature and then incubated overnight at 4 °C with primary antibodies. After washes with TBS, the blots were incubated for 1 h at room temperature with secondary HRP-linked anti-rabbit IgG antibodies diluted 1:50,000 (cat. #7074, Lot: 26, Cell Signaling Technology, Danvers, MA) or HRP-linked anti-mouse IgG antibodies diluted 1:50,000 (cat. #7076, Lot: 33, Cell Signaling Technology) and then washed again with TBS. The blots were developed using Immobilon Western Chemiluminescent HRP Substrate (cat. WBKLS0500, Merck KGaA) in an Azure c400 Imaging System (Azure Biosystems, Dublin, CA, USA). The Western blot images and band intensities were quantified using AzureSpor2.0 software (Azure Biosystems). Phosphorylated proteins of interest were detected with the following antibodies: phospho-Akt (Ser473) (D9E) XP^®^ rabbit mAb diluted 1:2000 (cat. #4060, Lot: 23 Cell Signaling Technology) and insulin receptor β (Tyr1150/1151) (19H7) rabbit mAb diluted 1:1000 (cat. #3024, Lot: 15, Cell Signaling Technology). The obtained data were normalized using an Akt (pan) (C67E7) rabbit mAb diluted 1:2000 (cat. #4691, Lot: 28, Cell Signaling Technology) or purified mouse anti-actin Ab-5 C4 antibody diluted 1:4000 (cat. 612656, Lot: 7033721, BD Bioscience, San Jose, CA, USA).

### 4.6. PTP1B Activity

The determination of PTP1B activity was performed by quantitatively measuring a tyrosine phosphopeptide (RRLIEDAEpYAARG) (cat. 12-217, Merck KGaA) and RR-src peptide (RRLIEDAEYAARG) (cat. BML-P308-0001, Enzo Life Sciences, Inc., Lausen, Austria) in reaction media (100 mM Hepes pH 7.5 (Cat A0302497, ACROS Organics™—Thermo Fisher Scientific), 20 mM EDTA (cat. 254045-500 g, Merck KGaA), 0.1% BSA, and 0.015% Brij-35 (Cat. 8019621000, Merck KGaA)) using the UPLC/MS/MS method. A Waters Acquity UPLC chromatograph (Waters UK, Elstree, UK) was coupled to a Waters Xevo TQ-S tandem mass spectrometer (Waters UK). Chromatographic separation was achieved on a Waters Acquity UPLC BEH C18 column (2.1 × 50 mm, 1.7 µm) (Waters UK) with a mobile phase consisting of a 0.1% formic acid (Cat. 84865.260, Thermo Fisher Scientific) aqueous solution (A) and acetonitrile (B) (Cat. 34851-2.5L-R, Merck KGaA). The mobile phase gradient programme was as follows: 0 min—5% B; 2 min—65% B; 3 min—98% B; 4 min—98% B; 4.2 min—5% B; and 6 min—5% B. The flow rate was 0.4 mL/min, and the column temperature was 40°C. The mass spectrometer was operated in positive ionization electrospray mode. Data acquisition was performed in MRM mode with the MS/MS transitions *m/z* 533.7 > 392.1 (RRLIEDAEpYAARG) and *m/z* 507.30 > 392.1 RRLIEDAEYAARG). The concentrations of the pTyr peptide and RR-src were measured against a four-point calibration curve. The calibration standards were prepared by spiking blank reaction media with the pTyr peptide and RR-src in a concentration range of 2.5—100 µM. The test samples were diluted 100-fold with a mixture of 0.1% formic acid in acetonitrile/water (1:1, *v*/*v*) and used for UPLC/MS/MS analysis.

### 4.7. Synthesis of Palmitoylcarnitine

**Figure 9:**
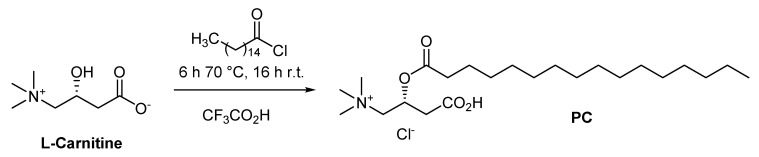
Synthesis of palmitoyl-L-carnitine hydrochloride (PC).

Palmitoyl-L-carnitine hydrochloride (PC) was synthesized from L-carnitine and palmitoyl chloride using a modified version of a protocol described by Nivet et al. [47]. Palmitoyl chloride (0.59 mL, 1.95 mmol) was added dropwise to a solution of L-carnitine (286 mg, 1.77 mmol) in trifluoroacetic acid (3 mL). The solution was stirred at 70 °C in the dark under argon for 4 h and then at room temperature for 16 h (Figure 9). The resulting mixture was cooled to room temperature, and diethyl ether (10 mL) was added. The white precipitate was filtered and washed with diethyl ether. The crude material was recrystallized from isopropanol (10 mL) to yield a white solid (475 mg, 61%).

NMR spectra were recorded at ambient temperature with a Varian 400 OXFORD NMR spectrometer (400 MHz). LC-MS was performed on an Acquity UPLC system (Waters UK) connected to a Micromass Quatro microTM API tandem mass spectrometer (Micromass—Waters UK) operating in ESI (electrospray ionization) positive ion mode and using an Acquity UPLC BEH HILIC column (1.7 μm, 2.1 × 100 mm) (Waters UK) with a gradient of 80–50% acetonitrile/10 mM ammonium acetate (pH 4). Elemental analysis was performed with a Carlo Erba EA 1108 instrument (CE Instruments Ltd., Hindley Green, UK). The melting point was measured using an OptiMelt melting point apparatus (Stanford Research Systems, Sunnyvale, CA, USA) and is reported uncorrected. Mp 157–159 °C. IR (KBr, cm-1): 3488 (O-H), 1748 (C=O). 1H–NMR (400 MHz, DMSO–d6, δ): 12.72 (s, 1H), 5.49–5.41 (m, 1H), 3.81 (dd, J = 14.2, 8.4 Hz, 1H), 3.65 (d, J = 14.2 Hz, 1H), 3.11 (s, 9H), 2.74–2.63 (m, 2H), 2.38–2.24 (m, 2H), 1.58–1.46 (m, 2H), 1.32–1.17 (m, 24H), 0.89–0.81 (m, 3H). 13C–NMR (100 MHz, DMSO–d6, δ): 172.1, 170.5, 67.1, 64.9, 53.0, 37.1, 33.6, 31.3, 29.04, 29.00, 28.9, 28.7, 28.4, 24.1, 22.1, 14.0. Anal. Calcd. for C23H46ClNO4: C, 63.35; H, 10.63; N, 3.21. Found: C, 63.11; H, 10.79; N, 3.12. [α]D20 -14,4 (c = 1.00, MeOH).

### 4.8. Other Materials Used

NovoRapid^®^ insulin aspart 100 units/mL (Novo Nordisk, Bagsværd, Denmark), sotrastaurin (Item No. 16726, Cayman Chemical, Ann Arbor, MI, USA) and (R)-(+)-etomoxir sodium salt (Cat. No. 4539, Tocris Bioscience) were diluted before use in sterile filtered PBS (137 mM NaCl, 2.7 mM KCl (cat. P9541, Merck KGaA), 10 mM Na_2_HPO_4_ (cat. 12695147, ACROS Organics™), 1.8 mM KH_2_PO_4_ (cat. 205920025, ACROS Organics™), pH 7.4). BVT948 (Item No. 16615, Cayman Chemical) and wortmannin (Cat. No. 1232, Tocris Bioscience) were dissolved in dimethyl sulfoxide (DMSO) (cat. D8418, Merck KGaA). BMOV was synthesized using the method previously described by Caravan el al. [48]

### 4.9. Statistical Analysis

The results are reported as the means ± standard deviations, and the statistical analysis of the data was performed using GraphPad Prism computer software (GraphPad, Inc., San Diego, CA, USA). The data distribution was determined using the Shapiro—Wilk normality test. The statistical significance of the experimental results was verified by one-way ANOVA followed by Dunnett’s multiple comparison test for normally distributed data. If the data were not normally distributed, the Kruskal–Wallis test followed by Dunn’s multiple comparison test was used. The results were considered statistically significant if the p-value was less than 0.05.

## 5. Conclusions

In conclusion, palmitoylcarnitine affects the insulin signalling pathway by activating PTP1B and inducing InsR Tyr1151 dephosphorylation, which consequently results in diminished Akt Ser473 phosphorylation. Palmitoylcarnitine also affects the downstream steps of the insulin signalling pathway and reduces Akt Ser473 phosphorylation independent of its effect on InsR phosphorylation. Inhibition of PP2A and PKCs does not affect the palmitoylcarnitine-induced dephosphorylation of Akt at Ser473, indicating different mechanisms of action.

## Data Availability

The datasets generated and analyzed during the current study are available from the corresponding author on reasonable request.

## Abbreviations

Akt	protein kinase B
BMOV	bis-(maltolato)-oxovanadium (IV)
BVT948	4-hydroxy-3,3-dimethyl-2*H*-benzo[*g*]indole-2,5(3*H*)-dione
CHO	CHO INSR 1284 cell line (ATCC^®^ CRL-3307™)
CPT1	carnitine palmitoyltransferase 1
DAG	diacylglycerol
InsR	insulin receptor
LCAC	long-chain acylcarnitine
PC	palmitoylcarnitine
PKC	protein kinase C
PP2A	protein phosphatase 2A
PTEN	phosphatase and tensin homolog
PTP1B	protein-tyrosine phosphatase 1B

## References

[1] Luo R.Z.T., Beniac D.R., Fernandes A., Yip C.C., Ottensmeyer F.P. (1999). Quaternary structure of the insulin-insulin receptor complex. Science.

[2] Feldhammer M., Uetani N., Miranda-Saavedra D., Tremblay M.L. (2013). Ptp1b: A simple enzyme for a complex world. Crit. Rev. Biochem. Mol. Biol..

[3] Sun J., Qu C., Wang Y., Huang H., Zhang M., Li H., Zhang Y., Wang Y., Zou W. (2016). PTP1B, A Potential Target of Type 2 Diabetes Mellitus. Mol. Biol..

[4] Scheid M.P., Marignani P.A., Woodgett J.R. (2002). Multiple Phosphoinositide 3-Kinase-Dependent Steps in Activation of Protein Kinase B. Mol. Cell. Biol..

[5] Whitman M., Downes C.P., Keeler M., Keller T., Cantley L. (1988). Type I phosphatidylinositol kinase makes a novel inositol phospholipid, phosphatidylinositol-3-phosphate. Nature.

[6] Dobrowsky R.T., Kamibayashi C., Mumby M.C., Hannun Y.A. (1993). Ceramide activates heterotrimeric protein phosphatase 2A. J. Biol. Chem..

[7] Eichmann T.O., Lass A. (2015). DAG tales: The multiple faces of diacylglycerol—Stereochemistry, metabolism, and signaling. Cell. Mol. Life Sci..

[8] Cong L.N., Chen H., Li Y., Zhou L., McGibbon M.A., Taylor S.I., Quon M.J. (1997). Physiological role of AKT in insulin-stimulated translocation of GLUT4 in transfected rat adipose cells. Mol. Endocrinol..

[9] Foster D.W. (2012). Malonyl-CoA: The regulator of fatty acid synthesis and oxidation. J. Clin. Investig..

[10] Ruderman N.B., Saha A.K., Vavvas D., Witters L.A. (1999). Malonyl-CoA, fuel sensing, and insulin resistance. Am. J. Physiol. Endocrinol. Metab..

[11] Makrecka-Kuka M., Liepinsh E., Murray A.J., Lemieux H., Dambrova M., Tepp K., Puurand M., Käämbre T., Han W.H., de Goede P. (2020). Altered mitochondrial metabolism in the insulin-resistant heart. Acta Physiol..

[12] Makrecka M., Kuka J., Volska K., Antone U., Sevostjanovs E., Cirule H., Grinberga S., Pugovics O., Dambrova M., Liepinsh E. (2014). Long-chain acylcarnitine content determines the pattern of energy metabolism in cardiac mitochondria. Mol. Cell. Biochem..

[13] Berezhnov A.V., Fedotova E.I., Nenov M.N., Kasymov V.A., Pimenov O.Y., Dynnik V.V. (2020). Dissecting cellular mechanisms of long-chain acylcarnitines-driven cardiotoxicity: Disturbance of calcium homeostasis, activation of Ca2+-dependent phospholipases, and mitochondrial energetics collapse. Int. J. Mol. Sci..

[14] Liepinsh E., Makrecka-Kuka M., Makarova E., Volska K., Vilks K., Sevostjanovs E., Antone U., Kuka J., Vilskersts R., Lola D. (2017). Acute and long-term administration of palmitoylcarnitine induces muscle-specific insulin resistance in mice. BioFactors.

[15] Aguer C., McCoin C.S., Knotts T.A., Thrush A.B., Ono-Moore K., McPherson R., Dent R., Hwang D.H., Adams S.H., Harper M.-E.M. (2014). Acylcarnitines: Potential implications for skeletal muscle insulin resistance. FASEB J..

[16] Blackburn M.L., Ono-Moore K.D., Sobhi H.F., Adams S.H. (2020). Carnitine palmitoyltransferase 2 knockout potentiates palmitate-induced insulin resistance in C2C12 myotubes. Am. J. Physiol. Endocrinol. Metab..

[17] Sommerfeld M.R., Müller G., Tschank G., Seipke G., Habermann P., Kurrle R., Tennagels N. (2010). In vitro metabolic and mitogenic signaling of insulin glargine and its metabolites. PLoS ONE.

[18] Gazdar A.F., Chick W.L., Oie H.K., Sims H.L., King D.L., Weir G.C., Lauris V. (1980). Continuous, clonal, insulin- and somatostatin-secreting cell lines established from a transplantable rat islet cell tumor. Proc. Natl. Acad. Sci. USA.

[19] Thompson K.H., Orvig C. (2006). Vanadium in diabetes: 100 years from Phase 0 to Phase I. J. Inorg. Biochem..

[20] Liljebris C., Baranczewski P., Björkstrand E., Byström S., Lundgren B., Tjernberg A., Warolén M., James S.R. (2004). Oxidation of Protein Tyrosine Phosphatases as a Pharmaceutical Mechanism of Action A Study Using 4-Hydroxy-3, 3-dimethyl-2H-benzo[g]indole-2,5(3H)-dione. J. Pharmacol. Exp. Ther..

[21] Vardatsikos G., Pandey N.R., Srivastava A.K. (2013). Insulino-mimetic and anti-diabetic effects of zinc. J. Inorg. Biochem..

[22] Kawakami Y., Nishimoto H., Kitaura J., Maeda-Yamamoto M., Kato R.M., Littman D.R., Rawlings D.J., Kawakami T. (2004). Protein kinase C βII regulates Akt phosphorylation on Ser-473 in a cell type- and stimulus-specific fashion. J. Biol. Chem..

[23] González-Rodríguez Á., Mas Gutierrez J.A., Sanz-González S., Ros M., Burks D.J., Valverde Á.M. (2010). Inhibition of PTP1B restores IRS1-mediated hepatic insulin signaling in IRS2-deficient mice. Diabetes.

[24] Zabolotny J.M., Haj F.G., Kim Y.B., Kim H.J., Shulman G.I., Kim J.K., Neel B.G., Kahn B.B. (2004). Transgenic overexpression of protein-tyrosine phosphatase 1B in muscle causes insulin resistance, but overexpression with leukocyte antigen-related phosphatase does not additively impair insulin action. J. Biol. Chem..

[25] Manning B.D., Cantley L.C. (2007). AKT/PKB Signaling: Navigating Downstream. Cell.

[26] Ravichandran L.V., Chen H., Li Y., Quon M.J. (2001). Phosphorylation of PTB1B at Ser50 by Akt impairs its ability to dephosphorylate the insulin receptor. Mol. Endocrinol..

[27] Peters K.G., Davis M.G., Howard B.W., Pokross M., Rastogi V., Diven C., Greis K.D., Eby-Wilkens E., Maier M., Evdokimov A. (2003). Mechanism of insulin sensitization by BMOV (bis maltolato oxo vanadium); Unliganded vanadium (VO4) as the active component. J. Inorg. Biochem..

[28] Zabolotny J.M., Kim Y.B., Peroni O.D., Kim J.K., Pani M.A., Boss O., Klaman L.D., Kamatkar S., Shulman G.I., Kahn B.B. (2001). Overexpression of the LAR (leukocyte antigen-related) protein-tyrosine phosphatase in muscle causes insulin resistance. Proc. Natl. Acad. Sci. USA.

[29] Lee H., Kim M., Baek M., Morales L.D., Jang I.S., Slaga T.J., DiGiovanni J., Kim D.J. (2017). Targeted disruption of TC-PTP in the proliferative compartment augments STAT3 and AKT signaling and skin tumor development. Sci. Rep..

[30] Cheng Y.S., Seibert O., Klöting N., Dietrich A., Straßburger K., Fernández-Veledo S., Vendrell J.J., Zorzano A., Blüher M., Herzig S. (2015). PPP2R5C Couples Hepatic Glucose and Lipid Homeostasis. PLoS Genet..

[31] Hein A.L., Seshacharyulu P., Rachagani S., Sheinin Y.M., Ouellette M.M., Ponnusamy M.P., Mumby M.C., Batra S.K., Yan Y. (2016). PR55α subunit of protein phosphatase 2A supports the tumorigenic and metastatic potential of pancreatic cancer cells by sustaining hyperactive oncogenic signaling. Cancer Res..

[32] Wu Y., Lu H., Yang H., Li C., Sang Q., Liu X., Liu Y., Wang Y., Sun Z. (2016). Zinc stimulates glucose consumption by modulating the insulin signaling pathway in L6 myotubes: Essential roles of Akt-GLUT4, GSK3β and mTOR-S6K1. J. Nutr. Biochem..

[33] Pandey N.R., Vardatsikos G., Mehdi M.Z., Srivastava A.K. (2010). Cell-type-specific roles of IGF-1R and EGFR in mediating Zn^2+^-induced ERK1/2 and PKB phosphorylation. J. Biol. Inorg. Chem..

[34] Wu W., Wang X., Zhang W., Reed W., Samet J.M., Whang Y.E., Ghio A.J. (2003). Zinc-induced PTEN protein degradation through the proteasome pathway in human airway epithelial cells. J. Biol. Chem..

[35] Li Y., Soos T.J., Li X., Wu J., DeGennaro M., Sun X., Littman D.R., Birnbaum M.J., Polakiewicz R.D. (2004). Protein kinase C θ inhibits insulin signaling by phosphorylating IRS1 at Ser1101. J. Biol. Chem..

[36] Evenou J.P., Wagner J., Zenke G., Brinkmann V., Wagner K., Kovarik J., Welzenbach K.A., Weitz-Schmidt G., Guntermann C., Towbin H. (2009). The potent protein kinase C-selective inhibitor AEB071 (sotrastaurin) represents a new class of immunosuppressive agents affecting early T-cell activation. J. Pharmacol. Exp. Ther..

[37] Leibiger I.B., Leibiger B., Moede T., Berggren P.O. (1998). Exocytosis of insulin promotes insulin gene transcription via the insulin receptor/PI-3 kinase/p70 s6 kinase and CaM kinase pathways. Mol. Cell.

[38] Bernal-Mizrachi E., Fatrai S., Johnson J.D., Ohsugi M., Otani K., Han Z., Polonsky K.S., Permutt M.A. (2004). Defective insulin secretion and increased susceptibility to experimental diabetes are induced by reduced Akt activity in pancreatic islet β cells. J. Clin. Investig..

[39] Aichler M., Borgmann D., Krumsiek J., Buck A., MacDonald P.E., Fox J.E.M., Lyon J., Light P.E., Keipert S., Jastroch M. (2017). N-acyl Taurines and Acylcarnitines Cause an Imbalance in Insulin Synthesis and Secretion Provoking β Cell Dysfunction in Type 2 Diabetes. Cell Metab..

[40] Liepinsh E., Makrecka-Kuka M., Makarova E., Volska K., Svalbe B., Sevostjanovs E., Grinberga S., Kuka J., Dambrova M. (2016). Decreased acylcarnitine content improves insulin sensitivity in experimental mice models of insulin resistance. Pharmacol. Res..

[41] McCoin C.S., Knotts T.A., Adams S.H. (2015). Acylcarnitines--old actors auditioning for new roles in metabolic physiology. Nat. Rev. Endocrinol..

[42] Pereyra A.S., Hasek L.Y., Harris K.L., Berman A.G., Damen F.W., Goergen C.J., Ellis J.M. (2017). Loss of cardiac carnitine palmitoyltransferase 2 results in rapamycin-resistant, acetylation-independent hypertrophy. J. Biol. Chem..

[43] Randle P.J., Garland P.B., Hales C.N., Newsholme E.A. (1963). The glucose fatty-acid cycle. Its role in insulin sensitivity and the metabolic disturbances of diabetes mellitus. Lancet.

[44] Hue L., Taegtmeyer H. (2009). The Randle cycle revisited: A new head for an old hat. Am. J. Physiol. Endocrinol. Metab..

[45] Liepinsh E., Makrecka-Kuka M., Volska K., Kuka J., Makarova E., Antone U., Sevostjanovs E., Vilskersts R., Strods A., Tars K. (2016). Long-chain acylcarnitines determine ischaemia/reperfusion-induced damage in heart mitochondria. Biochem. J..

[46] Makarova E., Makrecka-Kuka M., Vilks K., Volska K., Sevostjanovs E., Grinberga S., Zarkova-Malkova O., Dambrova M., Liepinsh E. (2019). Decreases in Circulating Concentrations of Long-Chain Acylcarnitines and Free Fatty Acids During the Glucose Tolerance Test Represent Tissue-Specific Insulin Sensitivity. Front. Endocrinol..

[47] Nivet J., Le Blanc M., Riess J. (1991). Synthesis and preliminary evaluation of perfluoroalkylacyl carnitines as surfactants for biomedical use. Eur. J. Med. Chem..

[48] Caravan P., Gelmini L., Glover N., Herring F.G., McNeill J.H., Rettig S.J., Setyawati I.A., Shuter E., Sun Y., Yuen V.G. (1995). Reaction Chemistry of BMOV, Bis(maltolato)oxovanadium(IV)—A Potent Insulin Mimetic Agent. J. Am. Chem. Soc..

